# Induction Chemotherapy Followed by Chemoradiotherapy With or Without Consolidation Chemotherapy Versus Chemoradiotherapy Followed by Consolidation Chemotherapy for Esophageal Squamous Cell Carcinoma

**DOI:** 10.3389/fonc.2022.813021

**Published:** 2022-05-23

**Authors:** Mingyue Xiang, Bo Liu, Guifang Zhang, Heyi Gong, Dali Han, Changsheng Ma

**Affiliations:** ^1^ Department of Radiation Oncology, Shandong Cancer Hospital and Institute, Shandong First Medical University and Shandong Academy of Medical Sciences, Jinan, China; ^2^ Department of Graduate, Shandong First Medical University and Shandong Academy of Medical Sciences, Taian, China; ^3^ Department of Medical Oncology, Shandong Cancer Hospital and Institute, Shandong First Medical University and Shandong Academy of Medical Sciences, Jinan, China

**Keywords:** esophageal squamous cell carcinoma, induction chemotherapy, concurrent chemoradiotherapy, consolidation chemotherapy, survival

## Abstract

**Objective:**

This study aimed to compare the efficacy and safety of induction chemotherapy followed by concurrent chemoradiotherapy (I-CCRT), induction chemotherapy followed by concurrent chemoradiotherapy and consolidation chemotherapy (I-CCRT-C), and concurrent chemoradiotherapy followed by consolidation chemotherapy (CCRT-C) for locally advanced esophageal squamous cell carcinoma (ESSC).

**Patients and Methods:**

Patients with locally advanced ESCC who underwent definitive chemoradiotherapy with cisplatin plus fluorouracil or docetaxel from February 2012 to December 2018 were retrospectively reviewed. Kaplan–Meier curve was used to estimate survival. Efficacy was assessed using RECIST, version 1.0. Prognosis factors were identified with Cox regression analysis.

**Results:**

Patients were treated with CCRT-C (*n* = 59), I-CCRT (*n* = 20), and I-CCRT-C (*n* = 48). The median follow-up duration was 73.9 months for the entire cohort. The ORR of the CCRT-C, I-CCRT, and I-CCRT-C groups was 89.8%, 70.0%, and 77.1%, respectively (*p* = 0.078). The median PFS in the CCRT-C, I-CCRT, and I-CCRT-C groups was 32.5, 16.1, and 27.1 months, respectively (*p* = 0.464). The median OS of the CCRT-C, I-CCRT, and I-CCRT-C groups was 45.9, 35.5, and 54.0 months, respectively (*p* = 0.788). Cox regression analysis indicated that I-CCRT-C and I-CCRT did not significantly prolong PFS and OS compared with CCRT-C (*p* > 0.05). Neutropenia grade ≥3 in CCRT-C, I-CCRT, and I-CCRT-C groups was 47.5%, 15%, and 33.3% of patients, respectively (*p* = 0.027).

**Conclusions:**

I-CCRT and I-CCRT-C using cisplatin plus fluorouracil or docetaxel regimen are not superior to CCRT-C in survival but seem to have less severe neutropenia than CCRT-C. Further randomized controlled studies are warranted.

## Introduction

Based on GLOBOCAN estimates, in 2018, esophageal cancer (EC) was the seventh most common cancer with approximately 572,000 newly diagnosed patients. It was also the sixth most common cause of cancer-related deaths, with 509,000 patient deaths (1). In China, EC is the third most diagnosed cancer with the fourth highest mortality rate (2). Unlike in western countries, esophageal squamous cell carcinoma (ESCC) is a predominant histopathological subtype of EC in China, comprising more than 90% of EC cases with a higher locoregional recurrence rate than adenocarcinoma (3).

Endoscopic resection is recommended as the standard option for intramucosal ESCC due to preservation of esophageal function and encouraging outcome (4). Surgical resection with neoadjuvant or adjuvant chemoradiotherapy is an important radical medical procedure for patients with resectable ESCC (5, 6). Unfortunately, half of ESCC patients are diagnosed at the locally advanced, unresectable stage associated with worsened prognosis (7). Even if medically fit for surgery, some patients with ESCC tend to receive radical chemoradiotherapy for the preservation of esophageal function.

Definitive chemoradiotherapy currently remains a treatment option for these patients who can to tolerate chemoradiation. Definitive chemoradiotherapy treatment options for ESCC include concurrent chemoradiotherapy (CCRT) (8), concurrent chemoradiotherapy followed by consolidation chemotherapy (CCRT-C) (9–11), induction chemotherapy followed by concurrent chemoradiotherapy (I-CCRT) (12, 13), and sequential chemoradiotherapy (SCRT) (14). Studies have demonstrated that CCRT confers a survival benefit for locally advanced ESCC compared with SCRT (14, 15). Therefore, CCRT and CCRT-C are recommended as the standard treatments for locally advanced unresectable ESCC (16). However, the outcome for ESCC patients receiving definitive CCRT remains poor, with 5-year overall survival (OS) rate of less than 20% (17).

Theoretically, adding induction chemotherapy before definitive CCRT has the potential to eradicate micrometastases, shrink tumor volume, and improve outcome (18), even reducing the radio-induced injury. A phase II study showed that I-CCRT with cisplatin-irinotecan is well-tolerated with a clinical complete response rate of 58.1% for EC (19). A retrospective study suggested that I-CCRT is superior to CCRT in OS and progress-free survival (PFS) for ESCC (18). However, the outcome and safety among I-CCRT, I-CCRT-C, and CCRT-C for patients with locally advanced ESCC has not been established.

Here, we conducted a retrospective study to compare the efficacy and safety of CCRT-C, I-CCRT, and I-CCRT-C with the chemotherapy regimen of cisplatin plus fluorouracil (PF) or docetaxel (DP) in locally advanced ESSC patients.

## Patients and Methods

Data of ESCC patients who received definitive CCRT-C, I-CCRT, and I-CCRT-C using the regimen of PF or DP were retrieved from our Medical Record System between February 2012 and December 2018 and analyzed. Variables included gender, age, Eastern Cooperative Oncology Group performance status (ECOG PS), serum levels of Cyfra 21-1 and carcinoembryonic antigen (CEA), tumor location, tumor length, T stage, N stage, M stage, differentiation, radiation technology, radiation dose, chemotherapy regimen, chemotherapy cycle, and treatment options. The inclusion criteria were as follows: age ≥18, ECOG PS ≤2, histopathologically confirmed squamous cell carcinoma, cT3-4N0M0/cT1-4N+M0 or cM1 (positive nonregional lymph nodes and irradiated during radiotherapy) in accordance with AJCC 7th edition, treated by 3DCRT/IMRT with radiation total doses ≥50 Gy using conventional fractionated radiotherapy, chemotherapy cycles ≥4, chemotherapy with PF or DP, no previous treatment, and no surgery after definitive chemoradiation. The exclusion criteria were as follows: underwent tumor resection before or after definitive chemoradiotherapy and changed chemotherapy regimens during definitive chemoradiotherapy. This study was approved by the ethics committee of our institute according to the Declaration of Helsinki. Patient informed consent was waived due to the nature of the retrospective study.

### Treatment Strategy

Concurrent chemoradiotherapy followed by chemotherapy included 1 to 6 cycles of chemotherapy after concurrent chemoradiotherapy (CCRT-C group). Induction chemotherapy followed by concurrent chemotherapy was defined as 1 to 6 cycles of chemotherapy delivered prior to concurrent chemoradiotherapy (I-CCRT group). Induction chemotherapy followed by concurrent chemoradiotherapy and consolidation chemotherapy included 1 to 4 cycles of chemotherapy followed by concurrent chemoradiotherapy and another 1 to 4 cycles of chemotherapy (I-CCRT-C group).

The chemotherapy regimens included the following: (i) cisplatin (60–80 mg/m^2^ on day 1) and docetaxel (60 mg/m^2^ on day 1); and (ii) cisplatin (75–100 mg/m^2^ on day 1) and fluorouracil (750–1,000 mg/m^2^ CIV 96 h on day 1). Chemotherapy was performed every 3 or 4 weeks, and the dosage was adjusted if necessary.

Patients lay on the examination bed of a big core CT fixed with a vacuum cushion. The radiotherapy was delivered using the 3DCRT or IMRT techniques. The plan was designed by Varian Eclipse or Pinnacle treatment planning system (TPS) with a 6-MV X-ray using 5, 7, or 9 coplanar radiated fields with elective or involved filed irradiation. The beam numbers and radiation directions were manually adjusted to optimize the plan. The gross tumor volume (GTV) was defined as the visible primary tumor (GTVp) and metastatic lymph nodes (GTVnd) detected by contrast-enhanced CT, PET/CT, and endoscopy. The clinical target volume of the primary tumor (CTVp) was defined as a 3.0-cm margin from the GTVp in up-down directions and a 0.5–0.6-cm margin in the posteroanterior and right–left directions. The clinical target volume of metastatic lymph nodes (CTVnd) was defined as a 0.5–0.6-cm margin from the GTVnd. The planning target volume (PTV) was generated from the CTVp and CTVnd with a 5-mm extended margin. The total radiation dose was delivered ≥50 Gy at 1.8 or 2 Gy per fraction, given once per day, 5 fractions per week. The PTV was covered with 95% of the prescription isodose line, and the volume receiving 104.5% of the prescription was limited to 5%. Dose-volume histograms (DVHs) were used to optimize target coverage and normal tissue sparing. The dose limitation for organs at risk (OARs) was defined as previously reported (20).

### Response Evaluation

The efficacy was evaluated by the Response Evaluation Criteria in Solid Tumors Version 1.0. PFS was defined as the period from the start of the anticancer treatment to the time of the first diagnostic progression or death or last follow-up. OS was defined from the start of the initial antitumor treatment to the date of death from any cause, regardless of disease status or last follow-up. The Common Terminology Criteria for Adverse Events Version 4.0 (CTCAE 4.0) was used to evaluate acute toxicities including leukocytopenia, neutropenia, anemia, thrombocytopenia, transaminase, bilirubinemia, and nausea/vomiting. Patients were followed up every 1 to 3 months after completion of chemotherapy for the first 2 years and every 6 to 12 months thereafter.

### Statistical Analyses

The Chi-square test or Fischer’s exact test was used to compare the difference for categorical variables. One-way ANOVA was used for continuous variables. A *p*-value reaching <0.05 was further compared using the rcompanion package for categorical variables or the LSD test for continuous variables. Survival was calculated using the Kaplan–Meier curve and compared by the log-rank test. Univariable and multivariable Cox regression analyses were used to identify the independent prognostic factors. Statistical analyses were performed using SPSS version 26 (IBM Corporation, USA) or R-3.6.3. The survival figure was delineated using GraphPad Prism 7.0 (GraphPad Software, USA). A *p*-value <0.05 was considered statistically significant.

## Results

### Patient Characteristics

A total of 127 patients treated with definitive chemoradiotherapy from February 2012 to December 2018 were analyzed in this study. Patients (59 of 127) were treated with CCRT-C, 20 with I-CCRT, and 48 with I-CCRT-C. The median tumor length in the CCRT-C, I-CCRT, and I-CCRT-C groups was 4.8, 4.6, and 5.7 cm, respectively (*p* = 0.031). *Post hoc* multiple comparisons showed that the I-CCRT-C group had longer primary tumors than CCRT-C (*p* = 0.023) and I-CCRT (*p* = 0.031). There were no significant difference in tumor length between CCRT-C and I-CCRT (*p* = 0.608). In the CCRT-C, I-CCRT, and I-CCRT-C groups, 49.2%, 75.0%, and 75.0% patients received the chemotherapy regimen of DP, respectively (*p* = 0.011). The CCRT-C group had more patients who received DP compared with I-CCRT-C (*p* = 0.034). There was no significant difference in the chemotherapy regimen between CCRT-C and I-CCRT (*p* = 0.120). The I-CCRT group also had a similar chemotherapy regimen to I-CCRT-C (*p* = 1.000). In total, 78.0%, 75.0%, and 31.3% were treated with 4 or 5 chemotherapy cycles in the CCRT-C, I-CCRT, and I-CCRT-C groups, respectively (*p* = 0). The I-CCRT-C group had fewer chemotherapy cycles than the CCRT-C (*p* = 0) and I-CCRT groups (*p* = 0.004). Meanwhile, the I-CCRT and I-CCRT groups had similar chemotherapy cycles. There were no significant differences in gender, age, ECOG PS, CEA, Cyfra 21-1, differentiation, T stage, N stage, M stage, radiation technology, and radiation dose among groups (*p* > 0.05). Detailed patient characteristics are summarized in **Table 1**.

**Table 1:** Characteristics of patients [*n* (%)].

Covariant	CCRT-C (*n* = 59)	I-CCRT (*n* = 20)	I-CCRT-C (*n* = 48)	*p-*value
Gender				0.101
Male	45 (76.3)	17 (85.0)	44 (91.7)	
Female	14 (23.7)	3 (15.0)	4 (8.3)	
Age (year)	59.2 ± 7.6	59.9 ± 6.2	61.8 ± 7.6	0.193
ECOG PS				0.231
0	35 (59.3)	9 (45.0)	21 (43.8)	
1	24 (40.7)	11 (55.0)	27 (56.2)	
Tumor location				0.218
Cervical or upper	37 (62.7)	11 (55.0)	22 (45.8)	
Middle or lower	22 (37.3)	9 (45.0)	26 (54.2)	
Tumor length (cm)	4.8 ± 1.9	4.6 ± 1.4	5.7 ± 2.4	0.031
CEA (ng/ml)				0.185
<3.4	43 (72.9)	18 (90.0)	40 (83.3)	
≥3.4	16 (27.1)	2 (10.0)	8 (16.7)	
Cyfra 21-1 (ng/ml)				0.089
<3.3	45 (78.0)	16 (80.0)	29 (60.4)	
≥3.3	13 (22.0)	4 (20.0)	19 (39.4)	
Differentiation				0.157
High	26 (44.1)	8 (40.0)	29 (60.4)	
Poor-middle	33 (55.9)	12 (60)	19 (39.6)	
T stage				0.363
T1–2	4 (6.8)	3 (15.0)	7 (14.6)	
T3–4	55 (93.2)	14 (85.0)	41 (85.4)	
N stage				0.162
N0	14 (23.7)	1 (5.0)	8 (16.7)	
N+	45 (76.3)	19 (95.0)	40 (83.3)	
M stage				0.230
M0	52 (88.1)	15 (75.0)	37 (77.1)	
M1	7 (11.9)	5 (25.0)	11 (22.9)	
Technology				0.927
3DCRT	20 (33.9)	7 (35.0)	18 (37.5)	
IMRT	39 (66.1)	13 (65.0)	30 (62.5)	
Dose (Gy)				0.075
<60	23 (39.0)	4 (20.0)	10 (20.8)	
≥60	36 (61.0)	16 (80.0)	38 (79.2)	
Regimen				0.011
DP	29 (49.2)	15 (75.0)	36 (75.0)	
PF	30 (50.8)	5 (25.0)	12 (25.0)	
Cycles				0
4–5	46 (78.0)	15 (75.0)	15 (31.3)	
6–8	13 (22.0)	5 (25.0)	33 (68.7)	

### Efficacy

The treatment response rate of CCRT-C, I-CCRT, and I-CCRT-C is summarized in **Table 2**. The objective response rate (ORR) in the CCRT-C, I-CCRT, and I-CCRT-C groups was 89.8%, 70.0%, and 77.1%, respectively (*p* = 0.078). The disease control rate (DCR) was 93.2%, 75.0%, and 100%, respectively (*p* = 0.009). I-CCRT-C had significantly higher DCR than I-CCRT (*P* = 0.006). However, CCRT-C had comparable DCR with I-CCRT (*p* = 0.106) and I-CCRT-C (*p* = 0.185).

**Table 2:** The treatment response rates among groups [*n* (%)].

Response	CCRT-C	I-CCRT	I-CCRT-C
Complete response	8 (13.6)	1 (5.0)	2 (4.2)
Partial response	45 (76.3)	13 (65.0)	35 (72.9)
Stable disease	2 (3.4)	1 (5.0)	11 (22.9)
Progression disease	4 (6.8)	5 (25.0)	0 (0)

### Survival

**Figure 1:**
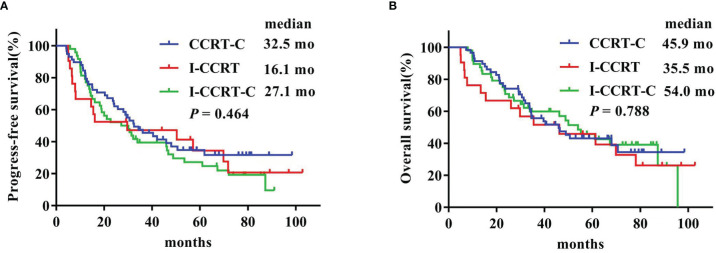
Kaplan–Meier curves of progress-free survival **(A)** and overall survival **(B)**.

The latest follow-up was in March 2021. The median follow-up duration was 73.9 months for the entire cohort. The Kaplan–Meier survival curves are shown in **Figure 1**. The median PFS in the CCRT-C, I-CCRT, and I-CCRT-C groups was 32.5 (95% confidence interval (CI): 20.3, 44.7), 16.1 (95% CI: 0, 47.0), and 27.1 (95% CI: 14.2, 40.0) months, respectively (*p* = 0.464) (**Figure 1A**). The median OS of the CCRT-C, I-CCRT, and I-CCRT-C groups was 45.9 (95% CI: 29.0, 62.8), 35.5 (95% CI: 1.4, 69.5), and 54.0 (95% CI: 38.5, 69.5) months, respectively (*p* = 0.788) (**Figure 1B**).

### Prognostic Factors

The univariate and multivariate Cox regression analyses of prognostic factors for PFS are shown in **Table 3**. In the univariable Cox regression analysis, age, ECOG PS, CYFRA 21-1, tumor differentiation, and M stage were potential prognostic factors for PFS. In the multivariable model using the Enter method, ECOG PS 1 [HR: 1.62 (95% CI: 1.01–2.61), *p* = 0.045], middle or poor differentiation (HR: 2.30 (95% CI: 1.47–3.60), *p* = 0), and M1 stage [HR: 1.72 (95% CI: 1.02–2.90), *p* = 0.44] were also associated with shorter PFS.

**Table 3:** Univariate and multivariate Cox regression of prognostic factors for PFS.

Covariant	Univariate Cox regression		Multivariate Cox regression	
	HR (95% CI)	*p*-value	HR (95% CI)	*p*-value
Gender female vs. male	0.77 (0.42, 1.38)	0.375		
Age	1.03 (1.00, 1.06)	0.032	1.03 (0.99, 1.06)	0.102
ECOG PS 1 vs. 0	2.09 (1.37, 3.18)	0.001	1.62 (1.01, 2.61)	0.045
CEA ≥3.4 vs. <3.4	0.91 (0.54, 1.54)	0.726		
Cyfra 21-1 ≥3.3 vs. <3.3	1.57 (1.01, 2.42)	0.045	1.38 (0.88, 2.16)	0.165
Tumor location				
Middle/lower vs. cervical/upper	1.25 (0.83, 1.90)	0.288		
Differentiation				
Middle or poor vs. high	2.26 (1.48, 3.45)	0	2.30 (1.47, 3.60)	0
Tumor length	0.97 (0.87, 1.07)	0.512		
T stage T3–4 vs. T1–2	1.75 (0.81, 3.78)	0.156		
N stage N+ vs. N0	1.30 (0.75, 2.28)	0.348		
M stage M1 vs. M0	1.73 (1.03, 2.92)	0.038	1.72 (1.02, 2.90)	0.044
Technology IMRT vs. 3DCRT	0.92 (0.60, 1.41)	0.687		
Dose ≥60 vs. <60 Gy	1.06 (0.67, 1.70)	0.792		
Regimen PF vs. TP	1.30 (0.85, 1.99)	0.219		
Cycles 6–8 vs. 4–5	1.36 (0.90, 2.07)	0.142		
Treatment options				
I-CCRT vs. CCRT-C	1.26 (0.69, 2.31)	0.449		
I-CCRT-C vs. CCRT-C	1.32 (0.84, 2.07)	0.234		

Univariate and multivariate Cox regression analyses predicting OS are summarized in **Table 4**. The results indicated that age, ECOG PS, CYFRA 21-1, differentiation, and M stage were possible OS predictive factors. In the multivariable analysis, middle or poor differentiation [HR: 2.47 (95% CI: 1.51–4.03), *p* = 0] and M1 stage [HR: 1.98 (95% CI: 1.12–3.49), *p* = 0.019] were the independent adverse predictors for OS.

**Table 4:** Univariate and multivariate Cox regression in predicting OS.

Covariant	Univariate Cox regression		Multivariate Cox regression	
	HR (95% CI)	*p*-value	HR (95% CI)	*p*-value
Gender female vs. male	0.68 (0.35, 1.32)	0.258		
Age	1.03 (1.00, 1.06)	0.077	1.03 (0.99, 1.06)	0.146
ECOG PS 1 vs. 0	2.10 (1.32, 3.34)	0.002	1.62 (0.97, 2.71)	0.065
CEA ≥3.4 vs. <3.4	1.04 (0.59, 1.83)	0.899		
Cyfra 21-1 ≥3.3 vs. <3.3	1.59 (0.98, 2.60)	0.062	1.46 (0.89, 2.40)	0.138
Tumor location				
Middle/lower vs. cervical/upper	1.31 (0.83, 2.06)	0.244		
Tumor length	1.00 (0.90, 1.12)	0.948		
Differentiation				
Middle or poor vs. high	2.49 (1.55, 4.00)	0	2.47 (1.51, 4.03)	0
T stage T3–4 vs. T1–2	1.55 (0.67, 3.57)	0.303		
N stage N+ vs. N0	1.44 (0.77, 2.68)	0.251		
M stage M1 vs. M0	1.83 (1.05, 3.19)	0.033	1.98 (1.12, 3.49)	0.019
Technology IMRT vs. 3DCRT	0.96 (0.60, 1.53)	0.848		
Dose ≥60 vs. <60 Gy	1.06 (0.63, 1.76)	0.839		
Regimen PF vs. TP	1.45 (0.92, 2.29)	0.115		
Cycles 6–8 vs. 4–5	1.26 (0.80, 1.98)	0.324		
Treatment options				
I-CCRT vs. CCRT-C	1.24 (0.66, 2.33)	0.509		
I-CCRT-C vs. CCRT-C	1.02 (0.61, 1.68)	0.950		

### Adverse Events

A summary of adverse events related to definitive chemoradiotherapy is provided in **Table 5**. Overall, these treatment strategies were relatively well tolerated. Hematological and gastrointestinal toxicities were the most common. Neutropenia grade ≥3 was observed in 47.5% of the CCRT-C group, 15% of the I-CCRT group, and 33.3% of the I-CCRT-C group (*p* = 0.027). *Post hoc* comparison demonstrated that the CCRT-C group had a higher incidence of severe neutropenia than the I-CCRT group (*p* = 0.021). However, the I-CCRT-C group had comparable severe neutropenia with the CCRT-C (*p* = 0.201) and I-CCRT (*p* = 0.215) groups. There were no significant differences in leukopenia, anemia, thrombocytopenia, radiation esophagitis, radiation pneumonitis, cardiac disorders, nausea or vomiting, and esophageal mediastinal or esophagotracheal fistula among the groups. There were no treatment-related deaths.

**Table 5:** Adverse events related to definitive chemoradiotherapy [*n* (%)].

Grade	CCRT-C	I-CCRT	I-CCRT-C	*p*-value
**Hematological**				
Leukopenia				0.412
0–2	35 (59.3)	14 (70.0)	34 (70.8)	
3–4	24 (40.7)	6 (30.0)	14 (29.2)	
Neutropenia				0.027
0–2	31 (52.5)	17 (85.0)	32 (66.7)	
3–4	28 (47.5)	3 (15.0)	16 (33.3)	
Thrombocytopenia				0.488
0–2	51 (86.4)	18 (90.0)	45 (93.8)	
3–4	8 (13.6)	2 (10.0)	3 (6.2)	
Anemia				0.157
0–1	59 (100.0)	19 (95.0)	48 (100.0)	
2–4	0 (0)	1 (5.0)	0 (0)	
**Nonhematological**				
Radiation esophagitis				0.381
0–2	49 (83.1)	18 (90.0)	44 (91.7)	
3–4	10 (16.9)	2 (10.0)	4 (8.3)	
Radiation pneumonitis				0.858
0–2	57 (96.6)	19 (95.0)	45 (93.8)	
3–4	2 (3.4)	1 (5.0)	3 (6.2)	
Cardiac disorders				0.752
No	53 (89.8)	18 (90.0)	41 (85.4)	
Yes	6 (10.2)	2 (10.0)	7 (14.6)	
Nausea or vomiting				0.058
0–1	27 (45.8)	11 (55.0)	33 (68.8)	
2–3	32 (54.2)	9 (45.0)	15 (31.2)	
Fistula				0.753
No	53 (89.8)	18 (90.0)	45 (93.8)	
Yes	6 (10.2)	2 (10.0)	3 (6.2)	

## Discussion

To the best of our knowledge, our study is the first to report the outcomes and safety of definitive chemoradiotherapy with CCRT-C, I-CCRT, and I-CCRT-C in patients with advanced ESCC. Our findings suggested that I-CCRT and I-CCRT-C are not superior to CCRT-C in survival for patients with advanced ESCC, based on the chemotherapy regimen of DP and PF, whereas I-CCRT had less grade ≥3 neutropenia than CCRT-C. Based on the results, I-CCRT or I-CCRT-C has the potential to be a standard treatment option for locally advanced ESCC.

According to the NCCN Guidelines for Esophageal Cancer 2021 v1, the preferred definitive chemoradiotherapy for nonsurgical EC was either CCRT or CCRT-C. However, several studies suggested that the addition of induction chemotherapy prior to CCRT in locally advanced ESCC was feasible (**Table 6**). A multicenter phase II FFCD trial (19) reported that induction chemotherapy with cisplatin and irinotecan followed by CCRT without surgery for stages I–III EC resulted in CR of 58.1% and 1- and 2-year OS of 62.8% and 27.9%, respectively. Watkins et al. (21) reported that induction cisplatin and irinotecan followed by concurrent cisplatin, irinotecan, and radiotherapy for locally advanced esophageal cancer is tolerable with a 2-year OS of 42% and 2-year PFS of 9.2%. A prospective, multicenter phase I/II study (16) reported that induction chemotherapy with docetaxel, cisplatin, and fluorouracil followed by CCRT was tolerable, with a CR of 39.4%, the median PFS of 12.2 months, and the median OS of 26.0 months in unresectable locally advanced ESCC. Another phase I/II study (22) found that induction chemotherapy with irinotecan, folinic acid, 5-fluorouracil, and cisplatin followed by concurrent chemoradiation with cisplatin and irinotecan was tolerable, with clinical CR, 1-year OS and local regional PFS of 56%, 77%, and 59%. In addition, Luo et al. (18) reported that I-CCRT had significantly longer OS compared with CCRT (26.0 vs. 22.0 months). However, whether the outcome and safety of I-CCRT or I-CCRT-C are superior to CCRT-C has not been established.

**Table 6:** Published literatures of definitive I-CCRT for ESCC.

Author	Number of patients	Regimen	Response	Outcome	Severe neutropenia [*n* (%)]
Michel et al. (19)	43	Cisplatin/irinotecan	CR 58.1%	1-year OS 62.8%	NA
			PR NA	2-year OS 27.9%	
Watkins et al. (21)	53	Cisplatin/irinotecan	NA	2-year OS 42%	13 (28%)
				2-year PFS 9.2%	
Satake et al. (16)	33	Docetaxel/cisplatin/5-Fu	CR 39.4%	mPFS 12.2 months	24 (72%)
			PR 33.3%	mOS 26.0 months	
				3-year OS 40.4%	
Pöttgen et al. (22)	16	Irinotecan/folinic acid/5-Fu/cisplatin	CR 56%	1-year OS 77%	10 (62%)
			PR NA	2-year OS 53%	
				3-year OS 41%	
				5-year OS 29%	
Luo et al. (18)	85	Docetaxel/cisplatin	CR+PR50.6% (after induction therapy)	mOS 26.0 months	33 (38.8%)
				3-year OS 30.6%	

NA, non-available.

Our findings suggested that the I-CCRT and I-CCRT-C groups had similar ORR to that of the CCRT-C group (70.0% vs. 77.1% vs. 89.8%). Previous reports showed that patient with ESCC receiving I-CCRT resulted in an ORR of 72.7% (16), similar to the present study. Our study also found that I-CCRT-C had a significantly higher DCR than I-CCRT, indicating that adding chemotherapy after I-CCRT might improve the DCR.

Our findings showed that the I-CCRT and I-CCRT-C groups had similar PFS (median, 16.1 vs. 27.1 vs. 32.5 months) and OS (35.5 vs. 54.0 vs. 45.9) with that of the CCRT-C group, which is superior to that of the PRODIGE5/ACCORD17 trial (median PFS, 9.7 months in the FOLFOX group and 9.4 months in the fluorouracil and cisplatin group) with CCRT-C. The reason likely was that the radiation dose of the present study was different from the PRODIGE5/ACCORD17 trial. A larger meta-analysis (23) reported that CCRT with doses of ≥60 Gy for ESCC patients might improve locoregional control and survival compared with the standard-dose CCRT. More than half of the patients received the prescribed total dose of ≥60 Gy, whereas the PRODIGE5/ACCORD17 trial used the standard dose radiation (50 Gy) with a conventional fraction. A phase II randomized controlled trial (24) demonstrated that CCRT with the DP regimen had similar treatment responses (ORR, 84.4% in the DP group and 87.3% in the PF group), PFS (1- and 2-year PFS, 77.4% and 55.0% for the PF group and 78.8% and 69.4% for the DP group), and OS (the 1- and 2-year OS, 93.7% and 86.2% for the PF group and 87.3% and 69.1% for the DP group) with those using CCRT with the PF regimen as a first-line treatment for patients with ESCC. Additionally, our study also suggested that the chemotherapy regimen was not associated with PFS and OS in the Cox regression analysis.

Several prospective or retrospective studies reported that the incidence of grade ≥3 neutropenia ranged from 2.6% to 41% in EC patients who received I-CCRT with a two-drug regimen. A randomized phase II study suggested that the incidence of grades 3–4 neutropenia of preoperative I-CCRT using oxaliplatin/capecitabine or carboplatin/paclitaxel for resectable esophageal adenocarcinoma was 2.6% (1/38) and 21.4% [9/42] (*p* = 0.011) (25). Another randomized phase II trial demonstrated that the incidence of grade 3 or 4 neutropenia of I-CCRT and CCRT alone with DP in ESCC was 18.2% and 7.3%, respectively (*p* = 0.151) (26). There were no significant differences in rates of other grades 3–5 hematological adverse events between groups were observed (26). Simoni et al. (13) reported that the rate of neutropenia (grade ≥3) of I-CCRT as an intensive neoadjuvant protocol for patients with EC was 22.7% (27/119). A matched case–control study (18) reported that the rates of grade ≥3 neutropenia of I-CCRT and CCRT with DP in the treatment of ESCC were 32.9% (*n* = 28) and 23.5% (*n* = 20) (*p* = 0.173), respectively. Additionally, more toxicity would be observed when using triple drugs during I-CCRT. A prospective, multicenter phase I/II study reported that the incidence of severe neutropenia in I-CCRT with a triple-drug of docetaxel, cisplatin, and fluorouracil in unresectable locally advanced EC was 72% (*n* = 33) (16). Another small sample study showed that I-CCRT (induction chemotherapy with irinotecan, folinic acid, and 5-fuorouracil weekly and cisplatin every 2 weeks followed by CCRT with cisplatin and irinotecan) for ESCC had more serious neutropenia (62%). Our study demonstrated that the neutropenia grade ≥3 was observed in 15% of the I-CCRT group, whereas 47.5% of the CCRT-C group and 33.3% of the I-CCRT-C group (*p* = 0.027). Zhu et al. (24) reported that definitive CCRT with a DP regimen was associated with more severe hematological toxicities than with PF regimen, including neutropenia. In the present study, the I-CCRT group was associated with less severe neutropenia than that of the CCRT-C group, which used fewer DP regimen and chemotherapy cycles. Taken together, we inferred that I-CCRT had a lower incidence of severe neutropenia than CCRT-C. We interpreted that the induction chemotherapy before CCRT might have the potential to shrink tumor volume, leading to less radiation volume, which could result in less neutropenia.

However, several limitations also existed in the present study, which require mentioning. First, this was a retrospective study, which could have had an influence on the quality of the data and the selection of patients. Second, there were a relatively small number of patients among groups, especially in the I-CCRT group. Third, the basic characteristics of patients, including tumor length, chemotherapy regimen, and chemotherapy, were unbalanced among groups. However, these factors did not significantly affect the PFS and OS in Cox regression analysis, thus having a limited effect in the present study.

## Conclusions

Our study suggested that I-CCRT and I-CCRT-C using cisplatin plus fluorouracil or docetaxel regimens are not superior to CCRT-C in ORR, PFS, and OS for locally advanced ESCC. I-CCRT or I-CCRT-C seems to have less severe neutropenia than CCRT-C. I-CCRT and I-CCRT-C have the potential to be treatment options for selective locally advanced ESCC patients. Prospective, randomized controlled studies are warranted to verify the presented results.

## Data Availability Statement

The original data are available if requested. Further inquiries can be directed to the corresponding authors.

## Ethics Statement

This study was approved by the Ethics Committee of Shandong Cancer Hospital and Institute according to the Declaration of Helsinki. Patient informed consent was waived due to the nature of the retrospective study.

## Author Contributions

MX and BL: drafted, conceived, and designed the manuscript. GZ, HG, and DH: contributed to acquiring, analyzing, and interpreting data. CM: contributed to acquiring data and enhancing its intellectual content. All authors read and approved the final manuscript.

## Conflict of Interest

The authors declare that the research was conducted in the absence of any commercial or financial relationships that could be construed as a potential conflict of interest.

## Publisher’s Note

All claims expressed in this article are solely those of the authors and do not necessarily represent those of their affiliated organizations, or those of the publisher, the editors and the reviewers. Any product that may be evaluated in this article, or claim that may be made by its manufacturer, is not guaranteed or endorsed by the publisher.

## Funding

This work is supported by National Nature Science Foundation of China (81800156, 81974467), Shandong Province Key R&D Program (2018GSF118031), and Natural Science Foundation of Shandong Province (ZR2019MH136, ZR2017BH024).
